# Effect of different sintering process on flexural strength of translucency monolithic zirconia

**DOI:** 10.4317/jced.54749

**Published:** 2018-08-01

**Authors:** Niwut Juntavee, Surawut Attashu

**Affiliations:** 1 Department of Prosthodontics, Faculty of Dentistry, Khon Kaen University, Khon Kaen, Thailand; 2 Division of Biomaterials and Prosthodontics Research, Faculty of Dentistry, Khon Kaen University, Khon Kaen, Thailand

## Abstract

**Background:**

Sintering process is responsible for the strength of zirconia restoration. This study evaluated the effect of different sintering temperatures and sintered-holding times on flexural strength of translucency monolithic zirconia.

**Material and Methods:**

One hundred and thirty five zirconia bar specimens (width-length-thickness = 10×20×1.5 mm) were prepared from yttria-stabilized tetragonal zirconia polycrystalline (Y-TZP) ceramic and randomly divided into nine groups to be sintered at different temperatures [decreasing- (SD, 1350°C), regular- (SR, 1450°C), and increasing- (SI, 1550°C) sintering temperature] and different sintered-holding times [shortening- (HS, 60 min), regular- (HR, 120 min), and prolonged- (HP, 180 min) sintered-holding time]. Flexural strength was determined using three-point bending test in a universal testing machine at 1 mm/min crosshead speed. An analysis of variance (ANOVA) and Tukey’s multiple comparisons were used to determine for statistically significant difference of flexural strength (α=0.05). Weibull analysis was applied for survival probability, Weibull modulus (m), and characteristics strength (σo) of the flexural strength. The crystal sizes were microscopically examined using scanning electron microscope (SEM). The phase composition of zirconia was determined using X-ray diffraction (XRD).

**Results:**

The mean±sd (MPa), m, and σo of flexural strength were 1080.25±217.19, 5.54, and 1167.53 for SDHS, 1243.41±233.17, 5.19, and 1352.30 for SDHR, 1298.92±235.68, 6.24, and 1394.79 for SDHP, 1303.34±171.87, 8.40, and 1377.90 for SRHS, 1331.73±278.84, 5.31, and 1444.50 for SRHR, 1348.13±283.35, 5.32, and 1460.68 for SRHP, 1458.45±289.19, 4.51, and 1604.41 for SIHS 1581.34±190.56, 8.20, and 1675.21 for SIHR and, 1604.10±139.52, 12.57, and 1667.90 for SIHP. The flexural strength was significantly affected by altering sintering temperatures and holding times (*p*<0.05). Enlarging grain size and increasing t→m phase shifting related with raising temperatures and times.

**Conclusions:**

Increasing sintering temperature and prolonged sintered-holding time lead to enhancing flexural strength of translucency monolithic zirconia, and are suggested for sintering process to achieve durable restoration.

** Key words:**Flexural strength, monolithic zirconia, sintering temperature, sintered-holding time.

## Introduction

Successful prosthodontics reconstruction with fixed dental restoration needs to achieve aesthetics, biocompatibility, and sufficient strength for withstanding the stress from the physiologic masticatory function. Patients often request for metal-free restoration, which leads to ceramic being the restoration of choice for reconstruction. The increasing celebrity of all-ceramic materials as an alternative to metal-ceramic restorations is attributable to their excellent aesthetics, corrosion resistance, and biological compatibility (1). Nevertheless, the inherited brittle property and low tensile strength of conventional ceramic limits their long-term clinical success. Several new dental ceramics have been developed with improved strengths for withstanding masticatory function force and being used as long-span fixed dental restorations (2,3). Among contemporary ceramics, yttria-stabilized tetragonal zirconia polycrystalline (Y-TZP) ceramic has recently been innovated as another possible restorative material, owing to its excellent aesthetics, biological compatibility, less plaque accumulation, minimal thermal conductivity, in addition to respectable fracture toughness, and strength (1,4,5). A unique characteristic of Y-TZP, on account of a transformation toughening phenomenon, has been reported to be capable of efficient inhibition crack propagation (3,6). Zirconia is a polycrystalline ceramic, which lacks glass component and possesses in three forms comprising monoclinic (*m*), cubic (*c*), and tetragonal (*t*) forms. The classical pure zirconia exhibits in the monoclinic crystalline structure at room temperature, which is stable up to 1,170°C. Above this temperature, a phase transformation to the tetragonal crystalline structure occurs, which is stable up to 2,370°C; beyond that the cubic crystalline structure is derived (7). In order to stabilize the zirconia in its tetragonal phase at room temperature, some stabilizing oxides such as calcium oxide (CaO), magnesium oxide (MgO), cerium oxide (CeO2) and yttrium oxide (Y2O3) were added. The tensile stresses, which contemporize at a crack tip, will evoke the tetragonal phase to transform into the monoclinic phase, resulting in a localized volume expansion of 3% to 5%. This volumetric expansion induces compressive stresses at the crack tip to counteract with the external tensile stresses and interrupt crack propagation (8). However, excessive external tensile stresses may exceed the compressive stresses under the surface and around the tip of the crack, leading to eventual failure of the material (9,10). Although the phase transformation may initially increase the fracture resistance of zirconia, the material may deteriorate due to different sintering temperatures, and fatigue forces (11,12).

The zirconia restorations can be fabricated from the process of computer-aided design and computer-aided manufacturing (CAD-CAM) technology. The zirconia milling process can be performed using either a fully sintered or partial sintered zirconia blank. The milling of a fully sintered zirconia blank to the actual size of the restoration provides precise accuracy, as the technique requires no further sintering process, thus eliminating the sintering shrinkage of zirconia (13). However, this technique causes excessive wear of the milling bur and takes a long time (14). The other milling procedure, which utilizes a partial sintered zirconia blank is easily machinable, but it needs to be sintered further to achieve fully sintered zirconia restoration (15). The zirconia restoration needs to be designed in an enlarged dimension prior to the milling process, in order to compensate for linear sintering shrinkage of zirconia by approximately 15–30% (16). The heat for sintering furnace is transmitted to the material’s surface and reaches its core by thermal conduction to achieve a mature sintered zirconia. The sintering process comprises a heating, a sintering, and a cooling phase (17). The sintering process may be altered in order to optimize the properties of zirconia. Even though, CAD-CAM technology has reduced the clinical operation times significantly, the zirconia sintering procedure still takes several hours. Even though zirconia possesses decent mechanical property, its opaque white color and deficient translucency requires glassy ceramic veneering to achieve a natural appearing esthetics restoration (18). However, delamination or chipping of the ceramic veneering material has been described as a frustrating complication of the restorations (19). The non-veneered, and full-contoured, monolithic zirconia restorations have become increasingly popular in order to eliminate veneer cracking and use in patients with high risk of excessive masticatory loads (20). There are two types of monolithic zirconia materials; opaque and translucent zirconia. The opaque zirconia offers significantly greater strength and usually indicates for restorations in the posterior regions of the mouth. The translucent zirconia provides more natural esthetic appearance and usually comprises the grain size less than 500 nm, allowing for better optical translucency upon sintering to be used in either the posterior or anterior regions of the mouth. To increase the translucency for full-contour zirconia, some attempts, such as the modification in sintering process, fabrication processes and coloring techniques, have been applied, which may alter zirconia properties (21).

Strength is considered as the clinical potential versus limitation of a dental ceramic restoration (4). Flexural strength is generally indicated as a relevant and reliable method to assess the durability of ceramic material. Materials with high flexural strength afford restorations with less susceptibility to fracture (22). Altering sintering parameters influences the strength properties of zirconia frameworks. Some studies were attempted to shorten the zirconia sintering process by inducing rapid heating rate and lowering the sintered-holding time; however, they reported no significant affect on the strength of zirconia core (9,23). Change in the sintering parameters through either increasing the sintered-holding time or the sintering temperature resulted in achieving better translucency (10,24). However, sintering at an extremely high sintering temperature was described to decrease flexural strength, due to migration of yttrium particles to the grain boundaries (9). The variations in sintering process of zirconia can directly affect the microstructure and properties of zirconia (25,26). It was described that variation in sintered-holding time during sintering process may affect the grain size and growth of zirconia microstructure, possibly affecting the strength and translucency of zirconia (9,10,27). As the grain size enlarges, zirconia may turn into more vulnerable to spontaneous t- to m- phase transformations, which may engender a gradual strength alteration (28,29). Among several studies, which indicated the effect of the change in sintering period and temperature on the optical translucency, microstructure and strength of zirconia core ceramics; however, the effect of changing these parameters on the strength of translucency monolithic zirconia are still questionable (10,23,26). This study’s aim was to determine whether the sintering temperature and sintered-holding time of Y-TZP monolithic material affect the strength. The null hypotheses were that varied sintering temperature and sintered-holding time would not affect the flexural strength of translucency monolithic zirconia.

## Material and Methods

-Zirconia specimen preparation 

One hundred and thirty five (135) zirconia specimens were prepared in a bar shape at the dimension of 12 mm width, 25 mm length and 1.8 mm in thickness from partially sintered yttrium-stabilized zirconia blanks (Y-TZP, VITA YZ HT color®, Vita Zahnfabrik, Säckingen, Germany) by using a diamond-coated wheel (Isomet® 1000, Beuhler, Lake Buff, IL, USA), ground down with a silicon carbide abrasive paper until 2400 grit particles, and polished with 1 µm diamond suspension using a polishing machine (Ecomet®3 polisher, Beuhler, Lake Bluff, IL, USA) to achieve the required dimension. All zirconia bar specimens were cleaned in the ultrasonic cleanser (Vitasonic II, Vita Zahnfabrik, Säckingen, Germany) with distilled water for fifteen minutes, and then dried in the room temperature. All specimens were randomly distributed into six groups (15 bars per group) according to the combination of three different sintering techniques: decreasing sintering temperature (SD, 1350°C), regular sintering temperature (SR, 1450°C), and increasing sintering temperature (SI, 1550°C), and three different sintered-holding times: shortening sintered-holding time (HS, 60 minutes), regular sintered-holding time (HR, 120 minutes), and prolonged sintered-holding time (HP, 180 minutes). All specimens were sintered in a sinter furnace (inFire® HTC, Sirona Dental Systems GmbH, Bensheim, Germany) at the heating and cooling rate of 17°C/min. The final dimension of each bar specimen (10 mm width, 20 mm length and 1.5 mm thickness) was derived due to approximately 20% volumetric shrinkage after sintering process.

-Flexural strength tests

**Figure 1:**
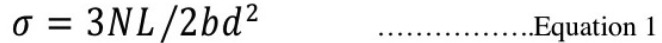
Equation 1.

All bar specimens were subjected to determination of three points flexural strength test (ISO 6872:2015 standard) in a universal testing machine (LR30/k, Lloyd, Leicester, England) at room temperature. The specimen was placed on a testing apparatus and compressively loaded at a crosshead speed of 1.0 mm/minute, until the specimen fracture occurred. The load at failure was recorded and calculated for the flexural strength by using equation 1, (Fig. 1).

In which: σ: flexural strength (MPa); N: fracture load (newton); L: distance between the supporting bars; b: width of specimen (mm), and d: thickness of specimen (mm).

-Microscopic examination of zirconia 

After sintering process, the surface of specimens in each group was serially polished with abrasive at grit 800, 2000, 4000, 6000, and finally with 1 µm diamond suspension in a polishing machine (Ecomet®3 polisher, Beuhler, Lake Bluff, IL, USA) and ultrasonically cleaned in distilled water for 15 minutes. Then, the specimens were dehydrated with acetone and dried at 50 ºC for 24 hours in the desiccator (Pyrex™ Fisher scientific Inc., Pittsburg, PA, USA). The specimens were coated with gold-palladium in sputter coater (K 500X, Emitech, Asford, United Kingdom) for 3 minutes at a current of 10 mA and vacuum 130 Torr. The surface topography and grain size were evaluated using a scanning electron microscope (SEM), (Hitachi S-3000N, Osaka, Japan) at magnifications of 30,000x.

-Crystalline structure analysis

**Figure 2:**
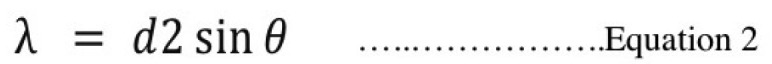
Equation 2.

The crystalline phases of monolithic zirconia were determined for the relative amount of monoclinic (m) and tetragonal (t) phase, using the X-ray diffraction (XRD, PANalytical, Empyrean, Almelo, Netherlands). The specimens were scanned with copper k-alpha (Cu Kα) radiation from the 2θ degree of 20–40o with 0.02o step size at every 2 seconds’ interval. The phase was analyzed in comparison to the known standard database of the joint committee on powder diffraction standards, and calculated for corresponding d-values using Bragg formula, as shown in Equation 2, (Fig. 2).

Where: λ is the X-ray wavelength (0.15418 nm for CuKa), d is normal distance of planes with the Miller indices (hkl), and θ is the Bragg angle.

**Figure 3:**
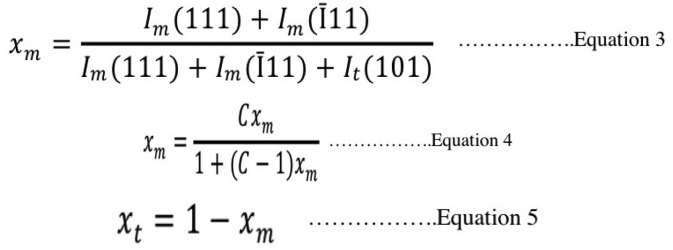
Equations 3,4,5.

The ratio of m- to t- phase was determined by the peaks’ intensities using X’Pert Plus software (Philips, Almelo, Netherlands). The mass fraction of m-phase to the total phase content was calculated from Garvie-Nicholson formula, shown in Equation 3, and further corrected for non-linearity using Toraya formula, shown in Equations 4 and 5 (30), (Fig. 3).

Where: Im and It: integral intensities of monoclinic and tetragonal phase

C: composition-dependent correction factor (C = 1.32)

Xt and Xm: the Toraya-corrected mass fraction of tetragonal and monolithic zirconia

-Statistical analysis

**Figure 4:**
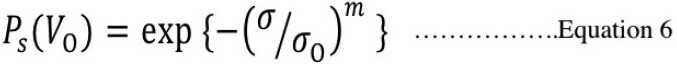
Equation 6.

The data was statistically analyzed using SPSS/PC Version 20 software (IBM, Armonk, NY, USA). An analysis of variance (ANOVA) was used to determine the significant differences in flexural strength upon different sintering temperatures and sintered-holding times. Post-hoc Tukey’s honest significant difference (HSD) multiple comparison was used to determine the difference between groups at 95% level of confidence. Weibull analysis was performed to evaluate the flexural strength’s reliability using Weibull++®statistics (ReliaSoft, Tucson, AZ, USA), and estimated the Weibull modulus (m) from Equation 6 and from a slope of the line plotted between ln{ln(1/Ps(Vo)} against m ln(σ/σo), (Fig. 4).

Where: Ps (Vo) is the probability of survival as the fraction of identical sample; Vo is the volume of the sample;

σ is the flexural strength; σo is the Weibull characteristic strength; and m is Weibull modulus.

## Results

**Figure 5:**
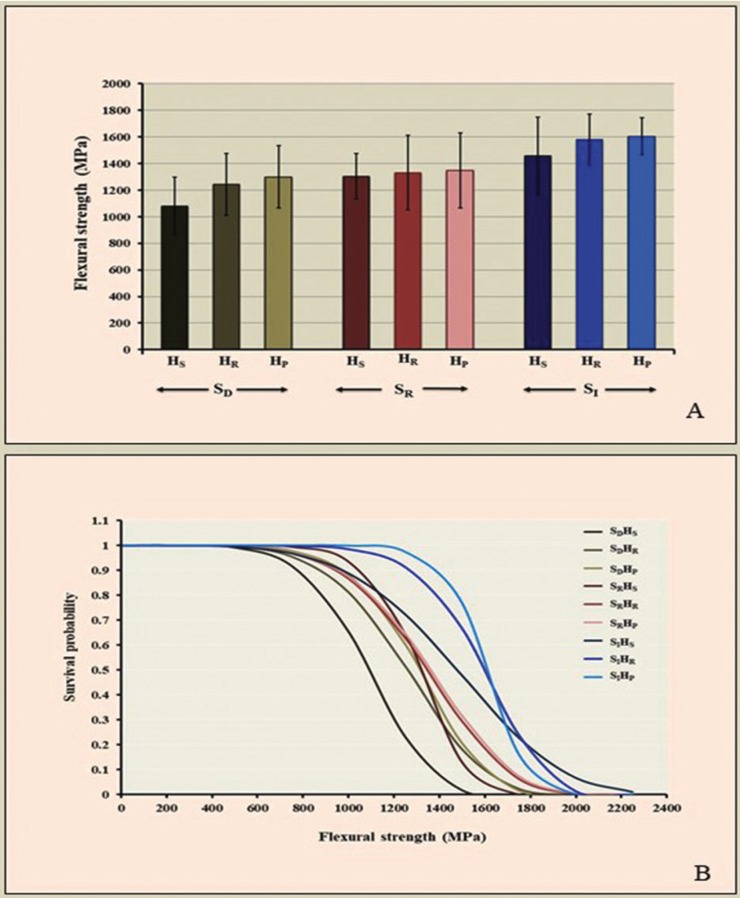
(A) Bar chart representing the comparison of flexural strength, and (B) line chart representing the comparison of Weibull survival probability of flexural strength for translucency monolithic zirconia, sintered at decreasing (SD), regular (SR), and increasing (SI) sintering temperature, with shortening (HS), regular (HR), and prolonged (HP) sintered-holding time.

The mean, standard deviation, 95% confidence interval, Weibull modulus (m), and characteristic strength (σo) for flexural strength for each group are presented in  Table 1 and Figure 5 (A). The highest flexural strength was demonstrated in the group SIHP (1604.10±139.52 MPa), followed by SIHR (1581.34±190.56 MPa), SIHS (1458.45±289.19 MPa), SRHP (1348.13±283.35 MPa), SRHR (1331.73±278.84MPa), SRHS (1303.34±171.78 MPa), SDHP (1298.92±235.68 MPa), SDHR (1243.41±233.17 MPa), and SDHS (1081.25±217.19 MPa), The evaluated results of the characteristic strength (σo, MPa) for SIHP, SIHR, SIHS, SRHP, SRHR, SRHS, SDHP, SDHR, and SDHS were 1667.90, 1675.21, 1604.90, 1460.68, 1444.50, 1377.90, 1394.79, 1352.30, and 1167.35 respectively, as presented in  Table 1.

**Table 1:**
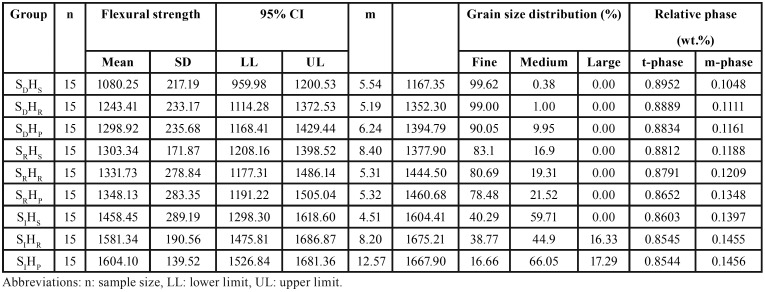
Mean, standard deviation (SD), 95% confidential interval (CI), Weibull modulus (m), characteristic strength (σo), percentage of grain size distribution (%), and relative phase content (wt.%) for flexural strength (MPa) of translucency monolithic zirconia, sintered at decreasing (SD), regular (SR), and increasing (SI) sintering temperature, with shortening (HS), regular (HR), and prolonged (HP) sintered-holding time.

An ANOVA indicated a statistically significant difference in flexural strength, because of varied sintering temperatures and sintered-holding times of zirconia sintering process (*p*<0.05), as shown in  Table 2. Post-hoc Tukey’s multiple comparisons indicated that sintering zirconia at an increasing sintering temperature resulted in significantly higher flexural strength, than at regular and decreasing sintering temperatures, while sintering zirconia at a decreasing sintering temperature resulted in significantly lower flexural strength than at regular sintering temperature (*p*<0.05), as presented in  Table 3. Post-hoc Tukey’s multiple comparisons indicated that prolonged sintered-holding time for zirconia resulted in significantly higher flexural strength, than at shortening sintered-holding time (*P*<0.05). However, there were no significant differences in flexural strength between prolonged- and regular-sintered holding time and between regular- and shortening sintered-holding time (*p*>0.05), as presented in  Table 3. Weibull analysis of flexural strength indicated Weibull modulus ranking from the highest to lowest, as for SIHP (12.57), SRHS (8.40), SIHR (8.20), SDHP (6.24), SDHS (5.54), SRHP (5.32), SRHR (5.31), SDHR (5.19), and SIHS (4.51); that was indicative of relative survival probability of flexural strength, as shown in Figure 5 (B) and  Table 1.

**Table 2:**
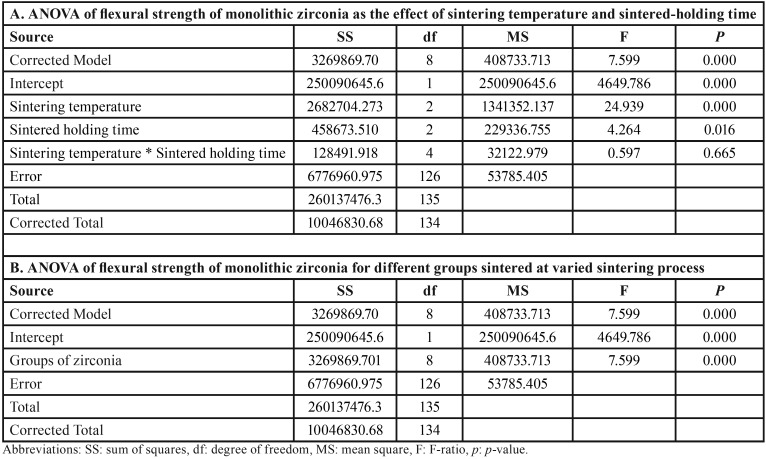
An analysis of variance (ANOVA) of flexural strength of translucency monolithic zirconia, sintered at decreasing (SD), regular (SR), and increasing (SI) sintering temperature, with shortening (HS), regular (HR), and prolonged (HP) sintered-holding time, indicated the effect of sintering temperature and sintered-holding time (A), and the effect of varied sintering process among the groups (B).

**Table 3:**
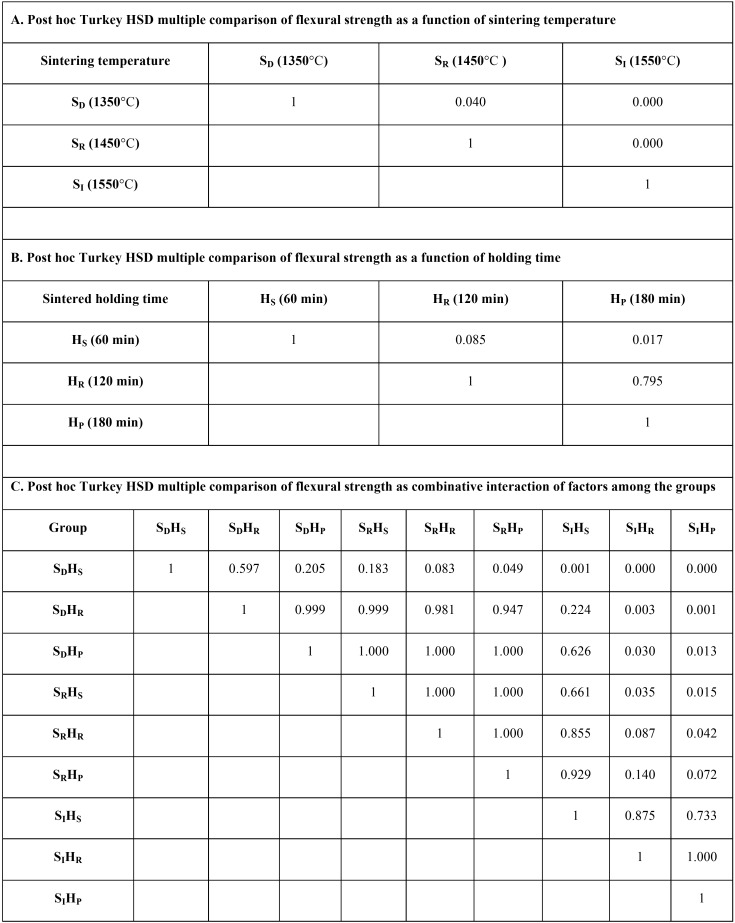
Post hoc Turkey HSD multiple comparisons of flexural strength of translucency monolithic zirconia, sintered at decreasing (SD), regular (SR), and increasing (SI) sintering temperature, with shortening (HS), regular (HR), and prolonged (HP) sintered-holding time, indicated the effect as a function of sintering temperature (A), holding time (B), and combinative interaction of factors among the groups (C).

**Figure 6:**
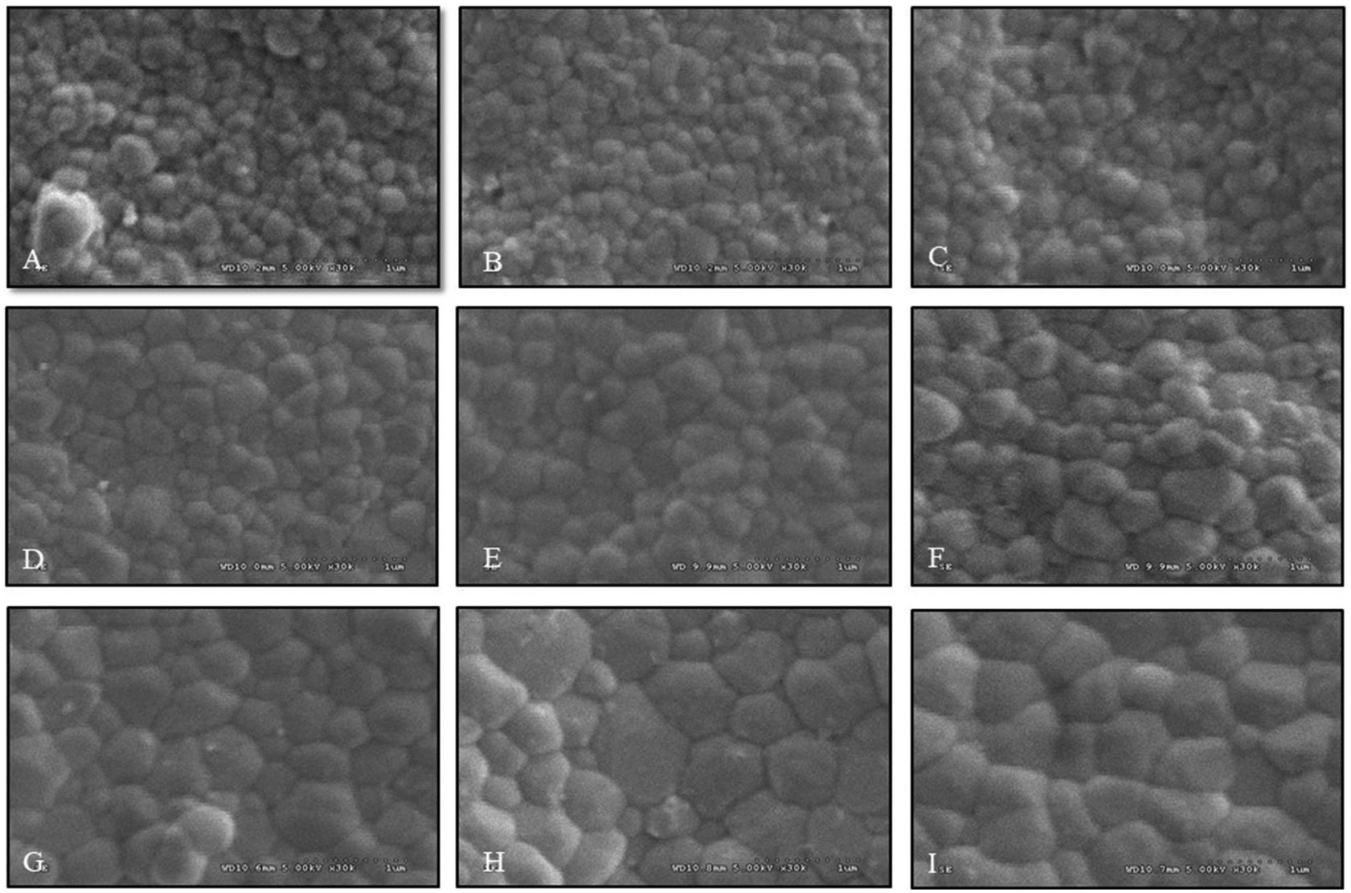
SEM photomicrographs indicated grain size and grain distribution of translucency monolithic zirconia, sintered at decreasing (A, B, C), regular (D, E, F), and increasing (G, H, I) sintering temperature, with shortening (A, D, G), regular (B, E, H), and prolonged (C, F, I) sintered-holding time at X30K magnification.

The SEM photomicrographs were used to observe zirconia grain size of monolithic zirconia. The difference in grain size was exhibited by the difference in sintering procedures, as present in  Table 1 and Figure 6. Sintering monolithic zirconia at decreasing sintering temperature exhibited crystal structures mostly in fine grains (0.1-0.4 μm). Increasing sintering temperature resulted in grain growth phenomenon and demonstrated an increase in medium grain sizes (0.5-0.8 μm) and large grain size (0.9-1.3 μm). The amount (%) of fine, medium, and large grain sizes were 99.62, 0.38, 0.00 for SDHS, 99.22, 0.78, 0.00 for SDHR, 90.05, 9.95, 0.00 for SDHP, 83.10, 16.90, 0.00 for SRHS, 80.69, 19.31, 0.00 for SRHR, 78.48, 21.52, 0.00 for SRHP, 40.29, 59.71, 0.00 for SIHS, 38.77, 44.90, 16.33 for SIHR, and 16.66, 66.05, 17.29 for SIHP group, respectively. The zirconia sintering process at increasing sintering temperature exhibited the amount of crystal structure in medium grain sizes, more than sintered, both in regular- and decreasing- sintering temperatures. It was also demonstrated that longer the sintered-holding time, the more grain growth was exhibited as present in Figure 6 and  Table 1. However, the prolonged sintered-holding time processes seemed to exhibit less effect on the growth of zirconia, when compared to the processes in raising sintering temperature. The photomicrographs also indicated defective integration of crystals at the grain boundary in the group, which sintered at decreasing sintering temperature and shortening sintered-holding time, while the crystalline structures exhibited densely compact crystal structures in the group that sintered at increasing sintering temperature and prolonged sintered-holding time.

**Figure 7:**
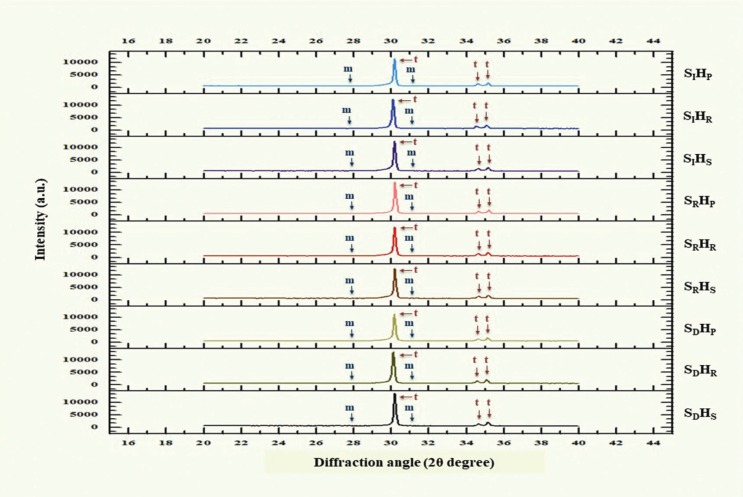
X-Ray diffraction analysis pattern of translucency monolithic zirconia, sintered at decreasing (SD), regular (SR), and increasing (SI) sintering temperature, with shortening (HS), regular (HR), and prolonged (HP) sintered-holding time.

The microstructure analysis of the specimens using XRD revealed that the peak positions for the spectra of the samples match the corresponding t- and m- forms as indicated from the XRD standard file of zirconium oxide (ZrO2). The XRD patterns revealed most of the crystal structure of t-phase with minor amount of m-phase in every group, as shown in Figure 7. The major peak intensity of t-phase was observed at the diffraction angle (2θ degree) of 30.177°, which corresponded to the Miller indices (hkl) crystallographic plane of the (101). The minor peaks of t-phase were observed at the diffraction angle of 34.607° and 35.172°, which matched to the crystallographic planes of (ī11) and (111) respectively. The m-phases were detected at the diffraction angle of 27.792° and 31.119°, which coincided with the crystallographic planes of (ī11) and (111), respectively. The t- and m-phase relative weight percentage (wt.%) concentrations were 0.8544, 0.1456 for SIHP, 0.8545, 0.1455 for SIHR, 0.8603, 0.1393 for SIHS, 0.8652, 0.1348 for SRHP, 0.8791, 0.1209 for SRHR, 0.8812, 0.1188 for SRHS, 0.8834, 0.1161 for SDHP, 0.8889, 0.1111 for SDHR, and 0.8952, 0.1048 for SDHS, as presented in Table 1. The relative amount of phase composition was relatively varied and associated with the sintering procedure of zirconia. The relative amount of m-phase increased as the zirconia was sintered at either higher sintering temperature or longer sintered-holding time. This indicated that the amount of phase composition shifting from t- to m- phase as increasing sintering temperature and lengthening sintered-holding time.

Ultimately, the result clearly indicated that altering sintering process through changing sintering temperature or duration of sintering time significantly affected flexural strength of monolithic Y-TZP. Sintering monolithic Y-TZP at high sintering temperature and long duration of sintered holding time resulted in higher flexural strength than sintered at low sintering temperature and short sintering time. The result was supported by the SEM indicating grain enlargement and XRD showing t→m phase shifting upon increasing sintering temperature and prolong sintering time.

## Discussion

This study indicated that flexural strength of translucency monolithic Y-TZP was affected by the alteration of sintering process, either sintering temperature or sintered-holding time. Thus, null hypothesis was rejected. Sintering monolithic zirconia at high sintering temperature and prolong sintered-holding time produced higher flexural strength than sintered at low sintering temperature and short sintered-holding time. This may relate with the maturation of crystal structures, the reduction in defective defects on the grain boundaries and the growth of grain sizes, achieved through either raising sintering temperature or the longer holding time, as supported by other studies (9,10,23). The increasing sintering temperature and prolonged sintered process determine the properties of monolithic zirconia by affecting both the microstructure and the crystalline phases of zirconia. The sintering process enables elimination of the inter-particle pores in the granular material by facilitating the atomic diffusion driven by capillary forces. As raising the sintering temperature or prolonged sintering time, the zirconia particles have higher capability of joining together, tending to minimize the pores on grain boundaries upon solid-state diffusion, and enabling increasing material density, which lead to enhancing strength of zirconia (9,26). This is a principal reason that longer holding time and higher sintering temperature groups achieve higher flexural strength than regular sintering programs. The results of this study are in agreement with the other studies (9,23,27,29).

The analysis of crystalline composition revealed that all groups of specimens contained mainly t-phase of zirconia grain. All specimens were completely sintered to achieve the t- and m- phase in their relative composition, with the absence of any transformation of phase, since no physical or thermal treatment was performed after each sintering. The varied sintering temperature and sintered-holding time affected the relative t- and m- phase combination of zirconia. The raising sintering temperature and lengthening sintered-holding time enable phase composition shifting from the t- to m- phase. Vise versa either lowering sintering temperature or shortening sintered-holding time indicated relatively less capability of phase shifting from t- to m- phase. The phase shifting phenomenon occurred as evidence supported from the grain size growth of zirconia seen on the SEM. The variation in sintering temperature seems to affect more the flexural strength, than the variation in sintered-holding time, as evidence supported from the alteration in grain size and the amount of t- to m- phase shifting indicated more effect upon altering sintering temperature. This t- to m- phase shifting phenomenon contributed to the increment of global residual compressive stresses in zirconia ceramic upon sintering process, leading to increasing crack inhibition, enforcing fracture resistance, and enhancing flexural strength of zirconia, as indicated in this study and supported by other studies (7,26,29).

The study suggested that altering sintering parameter of monolithic zirconia significantly affected the strength. It clearly indicated the variation in grain size and phase shift phenomenon between t- and m- phase, as the difference in sintering process, resulting in the strengthening of zirconia. Enhancing strength of translucency monolithic zirconia is possible upon either increasing sintering temperature or prolonged holding time. On the contrary, reducing sintering temperature or sintered-holding time can jeopardize flexural strength, which may lead to a perishable restoration.

## Conclusions

This investigation described the effect of sintering process on flexural strength of translucency monolithic Y-TZP. The study proved that flexural strength of monolithic Y-TZP was influenced by modification sintering temperature and duration of sintering time. Sintering monolithic zirconia at high sintering temperature and long sintering time rendered higher flexural strength than sintering at low temperature and short sintering time. Proper sintering process is extremely crucial to assure sufficient strength of monolithic Y-TZP. The sintering process at high sintering temperature and long duration of sintering time was suggested to maximized strength of translucency monolithic zirconia restoration.

## Clinical significance

Enhancing strength of translucency monolithic zirconia is possible, and can be achieved through altering sintering process. Either raising sintering temperature or extending sintered-holding time enables enhancing strength of monolithic translucency zirconia and is recommended for sintering procedure to derive durable zirconia restoration.
